# Development and piloting of a self-care psychoeducation program for mental health professionals

**DOI:** 10.3389/fpsyg.2026.1836750

**Published:** 2026-09-23

**Authors:** Aslıhan Cingirit, Begüm Serim-Yildiz, Tolga Erdogan

**Affiliations:** Department of Educational Sciences, TED University, Ankara, Türkiye

**Keywords:** burnout, psychoeducation program, qualitative research, self-care, trauma counselors

## Abstract

**Background:**

Trauma counselors are exposed to sustained emotional demands and indirect trauma, which may increase the risk of burnout, secondary traumatic stress, and compassion fatigue. Although self-care is recognized as an important component of ethical professional practice and has increasingly been examined as a potential protective approach, structured self-care interventions for in-service trauma counselors remain limited. This qualitative study aimed to develop a multidimensional self-care psychoeducation program for trauma counselors and to explore participants’ experiences and perceptions during the piloting of the program.

**Methods:**

The study was conducted in two interrelated phases within a qualitative research framework. In Study 1, a needs analysis was carried out with 11 trauma counselors to inform the development of a seven-session self-care psychoeducation program. In Study 2, the program was piloted with 17 trauma counselors over seven weeks. Data were collected through semi-structured interviews and session journal entries and analyzed using content analysis.

**Results:**

The needs analysis revealed prominent experiences of secondary traumatic stress and emotional exhaustion, as well as limited knowledge of self-care approaches despite the use of individual coping strategies. Participants described increased self-awareness of multidimensional self-care and reflected on cognitive-emotional, physical, spiritual, relational, and professional aspects of self-care throughout the pilot program. Participants also reflected on their perceptions of professional resilience and self-care habits.

**Conclusion:**

This study provides preliminary insights into trauma counselors’ experiences of participating in a multidimensional self-care psychoeducation program. Participants perceived group-based experiential learning, reflective practices, and peer support as valuable components of the program and described these experiences as contributing to their understanding of self-care strategies. These findings suggest that multidimensional self-care psychoeducation represents a promising area for further research. Future studies employing more rigorous designs are needed to examine the effectiveness and longer-term impact of such programs.

## Introduction

Throughout their professional careers, mental health professionals (MHPs) often work with clients who have experienced at least one traumatic event or are displaying symptoms of Post-Traumatic Stress Disorder (PTSD) (Howgego et al., 2005; da Silva et al., 2018). Due to the nature of trauma-focused practice, MHPs engage in intensive emotional labor, which involves the regulation of their own emotional responses while empathically attuning to clients’ distress (Hochschild, 1983; Grandey, 2000; Pearlman and Saakvitne, 1995a). Although emotional labor has been associated with positive professional outcomes, including job satisfaction, work effectiveness, overall well-being, and client satisfaction (Brunetto et al., 2014; Holman et al., 2008), it may also expose MHPs to considerable occupational risks due to sustained emotional demands (Brotheridge and Lee, 2002).

### Adverse experiences of MHPs

Indirect psychological impacts encountered by MHPs resulting from repeated exposure to clients’ traumatic experiences have been conceptualized using several related but distinct terms, such as burnout, vicarious trauma, and secondary traumatic stress (STS), and compassion fatigue (Figley, 1995; McCann and Pearlman, 1990).

Burnout, first described by Freudenberger (1974), refers to a state of physical, emotional, and mental exhaustion resulting from prolonged stress, high workload, and insufficient resources. It is commonly characterized by depersonalization, diminished personal accomplishment, and emotional exhaustion (Maslach and Jackson, 1981). Burnout among MHPs has been associated with anxiety and physical health problems at the individual level (Maslach and Leiter, 2016), as well as reduced job performance, increased errors, and workforce attrition at the professional and systemic levels (Figley, 1995; Schaufeli and Enzmann, 2020). Due to the sustained emotion regulation required in trauma-focused practice, MHPs appear to be at heightened risk, with reported prevalence rates ranging from 21% to 67% (Morse et al., 2012).

In addition to burnout, MHPs may experience trauma-related stress responses following repeated indirect exposure to clients’ traumatic experiences. Recognition that indirect exposure to trauma may result in trauma-related stress reactions has evolved across successive editions of DSM, from acknowledging exposure through learning about traumatic events affecting close others DSM-III (American Psychiatric Association [APA], 1980) to explicitly including repeated occupational exposure to aversive details of trauma in the DSM-5 (American Psychiatric Association [APA], 2022). Accordingly, STS refers to PTSD-like symptoms that may emerge following indirect exposure to another person’s traumatic experiences such as sleep and eating disturbances, as well as cognitive and emotional reactions including flashbacks, intrusive thoughts, and avoidance (Figley, 1993). Vicarious trauma, often discussed alongside STS, refers more specifically to cumulative changes in cognitive schemas concerning safety, trust, control, intimacy, and meaning that develop through repeated empathic engagement with trauma survivors (Pearlman and Saakvitne, 1995b). Although MHPs are often considered psychologically resilient (Skovholt and Trotter-Mathison, 2016), insufficient resilience may hinder the balance between personal needs and client care, increasing vulnerability to adverse psychological outcomes. Together, these constructs illustrate the range of psychological consequences associated with prolonged trauma-focused practice and underscore the importance of protective resources for mental health professionals. Recent qualitative evidence from psychological counselors and psychologists in Turkiye similarly documented diverse negative and positive effects of trauma-related work, as well as varied coping mechanisms and professional support needs (Yaşar and Tuzgöl-Dost, 2026).

In this regard, MHPs’ indirect occupational exposure to traumatic material may affect their professional competence, ethical responsibilities, personal well-being, and the effectiveness of therapeutic interventions. Accordingly, understanding MHPs’ cognitive, emotional, and professional responses to traumatic exposure is essential. Several theoretical frameworks have been proposed to explain how repeated exposure to clients’ traumatic experiences may influence MHPs psychological functioning. Constructivist Self-Development Theory explains that individuals construct their understanding of themselves and the world based on their experiences (Srdanovic, 2007), and that traumatic experiences may disrupt individuals’ fundamental beliefs about themselves and the world, which may result in emotional reactions, such as confusion and helplessness (Janoff-Bulman, 2002). Like their clients, MHPs may experience disruptions in beliefs about safety through vicarious experiences and encounter intense, difficult-to-process emotions. Such emotional strain may impair their empathic understanding (Harr, 2013), contribute to poor decision-making (Skovholt and Trotter-Mathison, 2016), weaken the therapeutic alliance, which is more critical than technique alone (Nissen-Lie et al., 2013), and ultimately reduce the effectiveness of the therapy (Bearse et al., 2013). The deterioration of such therapeutic skills may increase the risk of harm (Lawson, 2007), which is a core ethical principle in counseling (American Counseling Association [ACA], 2014).

In this context, ongoing exposure to traumatic material highlights the emotional and psychological burdens inherent in the work of MHPs. Despite the “cost of caring” (Figley, 1995, p.1), MHPs continue their work with an altruistic commitment (Stamm, 2005). The Etiological Model (Figley, 1995) addresses the psychological impact of working with trauma survivors by describing a continuum ranging from compassion satisfaction to compassion fatigue. While compassion satisfaction denotes the positive emotions derived from helping others, prolonged empathic engagement with trauma survivors may also contribute to increased stress and compassion fatigue over time (Turgoose and Maddox, 2017). Compassion fatigue is often conceptualized as an umbrella construct encompassing work-related emotional distress associated with vicarious traumatization, secondary traumatic stress, and burnout (Adams et al., 2006). It has been linked to PTSD-related reactions, including intrusive thoughts, avoidance, and arousal behaviors (Bride, 2004). Central to this model is empathy, which is essential for effective helping but may also increase vulnerability to emotional distress when MHPs become deeply immersed in their clients’ traumatic experiences (Livanou et al., 2024; Silveira and Boyer, 2015). This dual role of empathy underscores the importance of self-care practices and organizational support as protective resources to mitigate compassion-related distress (Ray et al., 2013).

Based on the Etiological Model, Stamm (2005) introduced the concept of Professional Quality of Life (ProQOL), which encompasses both the positive and negative outcomes of helping work and is associated with effective practice and professional retention (Killian, 2008). ProQOL reflects the balance between the costs and rewards of helping professions and is shaped by multiple individual and contextual factors (Stamm, 2002). Research indicates that ProQOL is shaped by a combination of work-related factors (e.g., high caseload, inadequate supervision, low workplace safety) (Meldrum et al., 2002), interpersonal resources (e.g., limited social support) (Stamm, 1999), and personal characteristics such as trauma history (Moulden and Firestone, 2007). Although findings remain mixed regarding the role of specific risk and protective factors, psychoeducation regarding trauma-related stress reactions and self-care practices has been consistently identified as important protective resources for supporting professional well-being. Accordingly, psychoeducational approaches (Figley, 1995) that increase awareness of trauma-related stress reactions while introducing practical self-care strategies (Saakvitne, 2002) have been associated with improved coping and professional well-being (Livanou et al., 2024). These findings provide a rationale for developing structured self-care psychoeducation programs for professionals working with trauma-exposed populations.

### Self-care as an intervention

Self-care, broadly defined as “caring for oneself” (Lee and Miller, 2013, p. 97), gained attention alongside the recognition of burnout as an occupational risk (Levin, 1976). Over time, the concept has expanded from individual health maintenance to a broader, multidimensional construct encompassing physical, emotional, cognitive, relational, and professional well-being, as well as social support (Dorociak et al., 2017) and strategies that reduce the emotional burden of helping work (Williams et al., 2010). Insufficient self-care among MHPs has been associated with reduced professional effectiveness (Welfel, 2015), diminished empathic capacity (McCann and Pearlman, 1990), burnout (La Mott and Martin, 2019) or STS (Owens-King, 2019) and eventually professional impairment (Sorenson et al., 2016). Despite being aware of the importance of self-care, MHPs often neglect it (Newell and MacNeil, 2010) due to barriers such as lack of time (Corey, 2013), perceiving it as a luxury (Norcross and Guy, 2018) or even a selfish activity (Sapienza and Bugental, 2000).

Importantly, self-care is not merely a personal preference but an ethical responsibility for MHPs (Wityk, 2003). Professional ethical frameworks and training standards explicitly emphasize the obligation to engage in self-care practices in order to maintain professional competence and prevent impairment (American Counseling Association [ACA], 2014; Council for Accreditation of Counseling and Related Educational Programs [CACREP], 2016). Despite being recognized as a protective factor against burnout and secondary traumatic stress, self-care remains insufficiently addressed through structured, experience-based interventions, particularly for in-service professionals working with trauma-exposed clients. Furthermore, relatively little attention has been paid to how self-care can be integrated across multiple domains of professional and personal functioning. Accordingly, there is a need for research that not only explores how self-care is understood and experienced, but also examines how structured psychoeducational approaches may facilitate the integration of self-care into professional practice by MHPs (Kissil and Niño, 2017; Park, 2010).

### The present study

Although self-care has received increasing attention in trauma-related professional contexts, structured self-care training specifically designed for in-service trauma counselors remains limited in Turkiye. Güler (2018) developed and implemented a self-care course for undergraduate counseling students, while Yaşar (2026) developed and evaluated a self-compassion-based psychoeducation program for mental health professionals. Previous programs targeting counseling students integrated mindfulness, reality therapy, and cognitive-behavioral techniques that improved participants’ self-care knowledge and attitudes. However, they primarily targeted counseling students or specific self-care approaches, leaving a need for multidimensional, needs-informed self-care psychoeducation specifically designed for in-service trauma counselors.

The present study aimed to (a) develop a theoretically grounded and needs-informed self-care psychoeducation program for in-service trauma MHPs working with trauma-exposed clients, and (b) examine participants’ experiences and perceptions of the program during its pilot implementation. This study conceptualizes self-care as a multidimensional process, which encompasses practices related to self-awareness (Coster and Schwebel, 1997), work–life balance (Bradley et al., 2013), adaptive coping (Campoli and Cummings, 2024; Simpson et al., 2019), physical health (Naczenski et al., 2017), social support (Barlow and Phelan, 2007), and meaning-making (Mason, 2016). By adopting a multidimensional perspective on self-care, this exploratory study aims to contribute to a deeper understanding of how trauma-exposed MHPs experience and integrate self-care within professional lives. The contribution of the present study lies in bringing together needs assessment, multidimensional self-care, and program development within a single qualitative framework specifically focused on in-service trauma counselors. Rather than examining a single self-care strategy or evaluating an established intervention, the study translates counselors’ identified needs into a structured psychoeducation program encompassing cognitive-emotional, physical, spiritual, relational, and professional domains. The two-stage design further connects program development with participants’ experiences during the pilot implementation, providing an initial empirical basis for refining the program and identifying potentially valuable program components for future evaluation.

## Materials and methods

### Study design overview

The present research was designed as a qualitative study consisting of two complementary studies conducted within a single research framework that aimed to capture both the developmental process of the program and participants’ subjective experiences. These two studies were conceptually and methodologically interrelated and jointly served the overarching aim of developing and examining a self-care psychoeducation program for trauma counselors. Study 1 focused on the development of the program, informed by a needs analysis and relevant literature, whereas Study 2 involved piloting the developed program to examine participants’ experiences and perceptions related to the program and self-care practices. The present research was intentionally limited to an exploratory qualitative pilot and did not include a subsequent main efficacy study; a controlled and longitudinal evaluation was considered beyond the scope of the present research. Together, these two studies allowed for an integrated examination of both the rationale underlying the program content and participants’ experiences during its implementation. Accordingly, the study addressed the following research questions:

(1) What are the challenges trauma counselors go through in their practices?
(2) How can a needs-informed self-care psychoeducation program be structured?
(3) What are trauma counselors’ experiences and perceptions regarding the self-care psychoeducation program?

Ethical approval for both Study 1 (the program development phase including needs analysis) and Study 2 (piloting the psychoeducation program) was obtained from the TED University Human Subjects Ethics Committee (Approval No: 2023-19). Informed consent was obtained from the participants prior to their involvement in each stage of research. The two studies were sequentially conducted, with findings from the needs analysis in Study 1 directly informing the content, structure, and implementation of the psychoeducation program piloted in Study 2.

### Participants

A purposive sampling method was used to recruit participants for both studies, with the inclusion criteria of being an active psychological counselor and having experience working with trauma-exposed clients. These inclusion criteria were applied consistently across both studies to ensure that participants could meaningfully contribute to the needs assessment and pilot implementation of the psychoeducation program. Given the exploratory qualitative design of the study, the aim was not statistical representation but to recruit information-rich participants capable of providing in-depth insights into self-care practices and trauma counseling experiences. Accordingly, sample adequacy was considered in relation to the information richness and data sufficiency required by the specific aims of each study rather than statistical representativeness or a predetermined numerical threshold.

#### Study 1. The development of the program

In Study 1, a needs analysis was conducted to inform the content and structure of the psychoeducation program. The needs analysis included interviews with 11 trauma counselors (female = 8, male = 3) with varying levels of experience (1.5 to 27 years), educational backgrounds (five with undergraduate degrees, five with master’s degrees, and one with a PhD), and ages (ranging from 25 to 58). These characteristics provided variation in the participants’ professional and educational backgrounds. The interview data yielded recurring and sufficiently detailed accounts of secondary traumatization responses, existing self-care practices, and perceived needs for self-care training, which directly informed the content and structure of the program.

#### Study 2. Piloting of the program

For Study 2, support was received from the Turkish Psychological Counselor and Guidance Association in promoting the psychoeducation program and the announcements were broadcast through their electronic platforms. Applicants completed a demographic information form and were screened for eligibility based on the predefined inclusion criteria. Ultimately, all participants (*N* = 17; female = 13, male = 4) who met these criteria were enrolled in the pilot program. The participants were aged between 23 and 52, and their counseling experience ranged from one to 30 years in the field of counseling. Participants represented a variety of professional settings, including schools, counseling centers, and private practice. The adequacy of the pilot data was further supported by repeated qualitative data collection across the seven-week implementation, with participants completing session journal entries at the end of each session. Thus, the pilot generated multiple, session-specific accounts of participants’ experiences rather than relying on a single point-in-time assessment.

### Measures and procedures

The study employed qualitative methods to address both the development and piloting of the self-care psychoeducation program for trauma counselors. Inductive content analysis was selected because the study aimed to systematically identify patterns and categories across participants’ experiences rather than to develop a formal theory or describe the essence of a lived experience, making this approach appropriate for addressing the exploratory research questions (Terry et al., 2017; Zhang and Wildemuth, 2009). Given the multidimensional nature of self-care and the absence of a comprehensive standardized instrument capable of assessing self-care across its multiple dimensions in Turkiye, qualitative data collection tools were considered appropriate to capture participants’ experiences and reflections in depth. Data collection and procedures were structured in alignment with the two complementary studies described below.

#### Study 1. Development of the program

Taking the multidimensional structure of self-care into account, several sequential stages were implemented in the development of the psychoeducation program specific to trauma counselors. The development process was guided by Taba’s (1962) inductive program development model, beginning with a needs analysis and proceeding through the specification of learning outcomes, organization of content, development of learning experiences, expert review, and pilot implementation. First, a needs analysis was undertaken to investigate psychological counselors’ prior experiences in the field of trauma, experiences of secondary trauma symptoms, and knowledge and application of self-care skills. The interview protocol was informed by relevant literature on trauma exposure, secondary traumatization, and self-care among mental health professionals. The purpose of the needs analysis was to identify participants’ perceived self-care needs, challenges encountered in trauma work, and expectations regarding a potential psychoeducation program. For this purpose, (a) an interview protocol was prepared, (b) the first draft was reviewed by experts and practitioners in trauma counseling and self-care to ensure validity, readability, and comprehension, (c) it was piloted on two trauma counselors and revised in light of the suggestions. In its final form, the interview form had six open-ended questions with three to six probes for each main question. Data obtained at this stage were used exclusively to guide the content, session structure, activities, and learning objectives of the program. Findings derived from the needs analysis are presented in the Results section.

Later, the results of the needs analysis and findings from relevant literature were used to develop the scope and content of the psychoeducation program. The linkage between the identified needs and the intervention components was established by integrating the needs analysis findings with the multidimensional conceptualization of self-care and relevant literature. Taba’s (1962) model served as the program development framework, whereas the theoretical rationale for the intervention components was informed by literature concerning cognitive-emotional self-care, physical well-being, self-compassion and meaning-making, relational support and boundaries, and professional self-care. Accordingly, identified needs were translated into learning outcomes and subsequently into session-specific content and activities. For example, reported difficulties with emotional regulation and recurrent thoughts informed learning objectives related to cognitive-emotional awareness and self-care planning, consistent with literature emphasizing awareness and self-regulation as dimensions of self-care among mental health professionals (Posluns and Gall, 2020; Richards et al., 2010). Reported physical stress responses informed objectives related to physical self-care and relaxation, consistent with literature highlighting physical well-being and relaxation as relevant self-care strategies for mental health professionals (Thériault et al., 2015). Needs concerning meaning, hope, and emotional relief informed the content of spiritual self-care, including self-compassion and meaning-oriented activities, consistent with literature linking self-compassion and meaning-making with well-being and adjustment (Nelson et al., 2018; Park, 2010). Relational needs informed activities addressing relationships, social support, and boundaries, reflecting literature emphasizing social support and relational resources in counselors’ coping and well-being (Thériault et al., 2015; Myers et al., 2012). Finally, professional stress and supervision-related needs informed activities addressing professional self-care, professional boundaries, work–life balance, and supervision, consistent with literature emphasizing supervision, professional development, and organizational support in addressing secondary trauma among mental health professionals (Harrison and Westwood, 2009; Thériault et al., 2015). Thus, Taba’s model guided the development process, while the needs analysis and relevant theoretical literature informed the rationale and content of the specific intervention components.

To this end, learning outcomes were developed and grouped into categories, the number and sequence of sessions were defined, and the content, materials, activities, delivery methods, and end-session evaluation practices were specified. To establish content validity, the draft psychoeducation program was reviewed by three experts, comprising two PhD-level counselor educators with expertise in trauma counseling and one PhD-level academic specializing in program development. They evaluated the program for its alignment with the intended goal of developing self-care skills among trauma counselors and the appropriateness of the selected methods, activities, materials, and evaluation practices. Revisions were subsequently made based on their feedback before the pilot implementation. In its final form, the psychoeducation program consisted of seven sessions, in each of which the instructor led the introduction and presentation in the first part, followed by participant-centered hands-on activities and session evaluations in the second part. Table 1 presents this needs-to-intervention linkage alongside the main activities, learning goals, and participants’ reported experiences during the pilot implementation.

**TABLE 1:** Summary of sessions.

Sessions	Theme	Needs identified	Learning goals	Main activities	Reported experiences
1	Introduction to self-care	Need for greater awareness of self-care and its role in trauma work; need for structured practical self-care guidance	Taking basic steps to create group dynamics and defining self-care and its dimensions	Group discussion; self-care mapping; strengths-based activities	Awareness; new perspectives; emotional reflection
2	Cognitive and emotional self-care strategies	Difficulties with emotional regulation and recurrent thoughts; need for strategies to recognize and manage cognitive and emotional needs	Recognizing the importance of cognitive and emotional self-care strategies and creating a self-care plan	Reflection; self-care planning; clay activity	Strength awareness; needs awareness; self-time; connectedness
3	Physical self-care strategies	Reported physical stress responses and insufficient attention to physical self-care	Identifying the importance of physical self-care strategies	Breathing; relaxation/body scan; physical self-care reflection	Body awareness; relaxation; intention to apply strategies
4	Spiritual self-care strategies	Needs related to meaning, hope, emotional relief, and developing a more compassionate relationship with oneself	Recognizing the significance of spiritual self-care strategies	Self-compassion; meaning/values reflection; letter writing; meditation	Relief; calmness; self-compassion; hope; occasional sadness
5	Relational self-care strategies	Need for peer/social support and greater awareness of relationships and interpersonal boundaries	Identifying the importance of relational self-care strategies	Relationship reflection; boundary exercise; painting activity	Relationship awareness; boundary awareness; happiness; anxiety/sadness
6	Professional self-care strategies	Professional stress; insufficient supervision/support; difficulties with professional boundaries and work–life balance	Recognizing the significance of professional self-care strategies	Case sharing; professional boundary reflection; goal setting; work–life balance	Professional awareness; new perspectives; positive affect; helplessness/inadequacy
7	Consolidation	Need to integrate self-care strategies into daily and professional life and sustain self-care practices	Evaluating the overall self-care strategies training process	Reflection; program evaluation; integration of self-care strategies	Behavioral change; cognitive awareness; emotional reflection

The reported experiences summarize themes identified in participants’ end-session journal entries in relation to the content and activities addressed in each session. These links are presented descriptively and reflect participants’ subjective reflections rather than causal effects of specific program components.

#### Study 2. Piloting of the program

In Study 2, the developed psychoeducation program was piloted. The intervention lasted for 7 weeks with an average of 2 h for each session. The sessions were conducted face-to-face at TED University. To document participants’ experiences during the implementation of the program, they were asked to complete the Session Journal Questionnaires at the end of each session. The session-specific journal data were subsequently used to examine participants’ reflections on the content and activities addressed in each session.

#### Session journal questionnaires

Session Journal Questionnaires were developed explicitly to capture participants’ session-specific experiences and reflections. For that purpose, seven distinct journal entries were prepared and completed at the end of each session. Each journal included open-ended questions designed to encourage participants to reflect on the session content, personal insights, perceived relevance, and anticipated application of self-care practices in their professional and personal lives.

Qualitative data obtained from the needs analysis (Study 1) and session journal entries (Study 2) were analyzed using inductive content analysis to explore patterns and meanings within participants’ experiences (Terry et al., 2017; Zhang and Wildemuth, 2009). Initially, all entries were read independently by two researchers for data familiarity. Meaningful units were then coded and grouped into broader categories that reflected recurring patterns across participants’ accounts. Coding decisions and category development were discussed and discrepancies were resolved through discussion and consensus. Finally, the findings were reported with illustrative statements extracted from participant entries. Participant confidentiality was ensured by providing excerpts with codes, where in Study 1 they were identified as DP# (Development Participant) and in Study 2 as PP# (Piloting Participant).

#### Trustworthiness

The present study utilized four essential criteria for assessing interpretive research: (a) credibility, (b) transferability, (c) dependability, and (d) confirmability to ascertain trustworthiness (Lincoln and Guba, 1985). Credibility was supported through member checking conducted at the end of the psychoeducation program. Participants were presented with a summary of the emerging findings and interpretations derived from their experiences and were invited to comment on whether these interpretations accurately reflected their experiences. Their feedback was considered during the refinement of the findings. In addition, prolonged engagement with participants throughout the seven-week program, with weekly sessions lasting approximately 2 h, facilitated the development of trust and contributed to the richness and credibility of the data. This research was primarily conducted through purposive sampling by focusing specifically on trauma-experienced psychological counselors in the field to enhance transferability. Transferability was further supported by providing contextual information about the participants and study setting, as well as by documenting the use of session journals and the systematic archiving of activities, materials, and session visuals. Dependability was enhanced through a systematic coding process involving two researchers who independently reviewed the data, discussed coding discrepancies, and reached consensus through iterative comparison. To ensure confirmability, all stages from data collection to analysis were documented, the psychoeducation program was reviewed by three experts, and the research design, methods, and materials were aligned with the research objectives. An audit trail documenting the stages of data collection, coding, category development, and interpretation was maintained throughout the study to enhance confirmability. Data triangulation was supported through the use of multiple qualitative data sources, including the needs-analysis interviews, session journal entries, and final participant reflections on the program.

#### Reflexivity

Reflexivity was maintained throughout the research process by acknowledging the researchers’ dual roles in program development, implementation, and qualitative analysis. The research team consisted of an experienced psychological counselor, a PhD-level counselor educator specializing in trauma counseling, and a PhD-level academic specialized in curriculum and instruction. Given their active involvement in developing and implementing the psychoeducation program, the researchers met regularly throughout the study to critically discuss emerging interpretations, reflect on potential assumptions, and revisit the original data whenever coding disagreements arose. Although the session journals were developed around predefined self-care domains that reflected the structure of the psychoeducation program, the coding process remained data-driven, allowing themes and categories to emerge inductively from participants’ accounts. The use of independent coding, discussion until consensus, member checking, and extensive participant quotations further supported reflexivity and minimized the influence of researcher assumptions on the interpretation of the findings.

In addition, given the research team’s dual roles as program facilitators, supervisors, and researchers, reflexivity was maintained throughout the implementation and analysis processes. One researcher facilitated the program, while a second researcher, who had a background in psychological counseling, co-led and supervised the implementation, and a third researcher with expertise in program development supervised the alignment between the program inputs, activities, and intended outputs. Following each session, the research team conducted detailed reflective evaluations of the implementation process, including consideration of the potential influence of facilitator–participant power differences, expectancy effects, and social desirability on participants’ responses. Particular attention was given to avoiding a hierarchical relationship that could encourage socially desirable or expectancy-consistent responses. During analysis, two researchers independently coded the qualitative data, returned to the original data when reviewing emerging codes and interpretations, and discussed discrepancies until consensus was reached. The interpretations and program-related outputs were also reviewed by the third researcher as an additional layer of reflexive scrutiny.

## Results

Results for the development of the program (Study 1) and the piloting of the program (Study 2) are reported separately below.

### Study 1

#### Needs analysis interview results

The analysis of the interviews with 11 counselors at the initial needs analysis stage of the program development cycle revealed themes illustrated in Figure 1.

**FIGURE 1:**
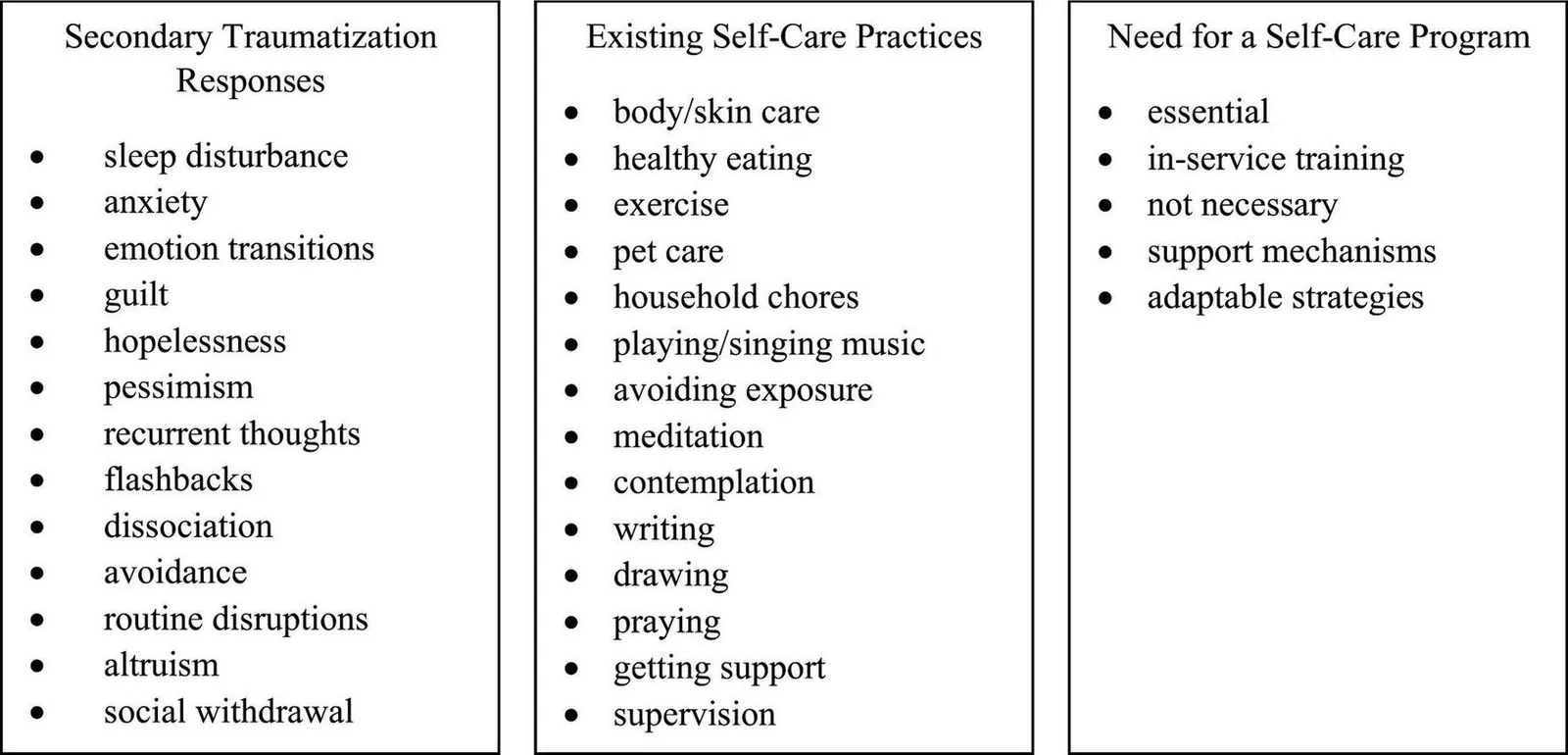
Themes for the needs analysis interviews.

##### Secondary traumatization responses

Participants talked about various secondary traumatization responses resulting from their work with trauma-exposed clients. These responses clustered into physical, emotional, cognitive, and behavioral domains. Table 2 summarizes the secondary traumatization responses identified in the needs analysis and the number of participants whose interview data were coded under each response category.

**TABLE 2:** Secondary traumatization responses reported by participants.

Domain	Secondary traumatization response	*n*
Physical	Sleep disturbance	10
	Anxiety-related somatic reactions	7
Emotional	Depressive mood / emotion transitions	9
	Guilt	7
	Hopelessness	5
	Pessimism	4
Cognitive	Recurrent thoughts	10
	Flashbacks	10
	Dissociative experiences	9
	Avoidance	10
Behavioral	Disruptions in daily routines	11
	Increased helping behaviors / altruism	9
	Social withdrawal	9

*n* represents the number of participants whose interview data were coded under each response category. Participants could be coded under multiple categories.

Most participants mentioned sleep disturbances and anxiety-related somatic reactions as a physical response. One of the participants stated, “When I first started working, I slept with the lights on every night for a week. I couldn’t sleep, actually. (DP4)” and another one recalled, “I actually had some problems with sleep for a while. There was a state of early awakening. (DP7)” Regarding anxiety, one of the counselors shared, “For example, lately I have noticed a delayed trauma symptom for a week or 10 days; when I go to bed at night, I wonder if there will be an earthquake. I feel like there will be a very severe earthquake. I can’t get out of it. (DP1).”

Participants also reported various emotional responses, including depressive mood, guilt, hopelessness and pessimism. One of the participants emphasized sudden emotional changes by expressing, “So, even though we are professionals, sometimes we really get stuck in a depressive mood, sometimes can’t get out of it, sudden transitions from one emotion to another. (DP2)” Referring to guilt, one counselor added, “The feeling of guilt I feel when my own needs are met while their needs are not. (DP10)” and addressing hopelessness, another participant recalled, “So there was hopelessness that covered all my humanity. (DP2)” Pessimism was another emotional response reported by the participants, as one of the counselors stated, “You could read the written statements of abused children. You could talk to them. And each time I saw a file or met a client, I always felt that it couldn’t get any worse. But I realized that it was too early to say that. My heart got darker each time I got home from work. (DP3)”

In addition to physical and emotional reactions, participants described cognitive and behavioral responses associated with repeated exposure to clients’ traumatic experiences. Cognitive responses included recurrent thoughts, flashbacks, dissociative experiences, and avoidance, whereas behavioral responses involved disruptions in daily routines, increased helping behaviors, and social withdrawal. When describing attempts to cope with recurrent thoughts, one counselor mentioned, “I usually find myself repeating the story in my mind over and over again. (DP11),” while another counselor talked about flashbacks by stating, “I have internalized the stories of my clients so much that, for example, their stories come to my mind like picture frames. (DP5)” One participant expressed dissociation by recalling, “While listening to the client, I feel like I’m breaking away and going somewhere else. (DP1)” and another participant emphasized avoidance by stating, “After the earthquake, I completely locked myself at home and I acted like nothing happened for a very long time… When I went to my mother’s house, I didn’t go to my grandfather’s grave, I didn’t enter his room for a very long time. (DP6).” Beyond these cognitive responses, participants also described behavioral changes affecting their daily functioning and interpersonal interactions. The behavioral responses identified in the interviews included disruptions in routines, altruism, and social withdrawal. One counselor’s statement exemplified disruption in routines as, “My routines were significantly affected. My sleep schedule was also part of those routines. (DP10),” another participant illustrated altruism by stating, “Suddenly, I found myself connected to the television and social media, trying to support in any way I could… Even from a distance, to provide support with whatever I had. (DP7),” and one counselor recalled social withdrawal by stating, “I started to avoid social interaction and communication. (DP6)”

##### Existing self-care practices

The analysis of interviews revealed that counselors were already practicing several self-care strategies across the physical, cognitive-emotional, spiritual, relational, and professional domains before the psychoeducation program. Physical self-care practices included activities aimed at maintaining physical well-being and reducing stress. Participants reported engaging in exercise, healthy eating, personal care routines, spending time with pets, and household activities. The following excerpts illustrate some of the physical self-care practices described by the participants: “I have 1.5-2-h shower sessions. I moisturize, exfoliate, apply creams, use hair masks, and spend time with fragrances. Physically, I need to feel good. (DP5)”

“I pay attention to regular eating, although it’s not always perfect. I avoid carbonated drinks and try not to consume carbohydrates. Especially during stressful periods, I am more cautious because I tend to eat more when stressed. (DP11)”

“I try to exercise for at least 15 min a day. Even though I’m tired, I feel good when I do it. (DP10)”

“Taking care of my cats, showing them love, spending time with them, and playing with them makes me feel good. (DP10)”

“House cleaning. It’s a powerful method to reduce my physical, emotional, and cognitive load. In that moment, all I think about is cleaning. It’s as if my thoughts are also cleansed when those areas are cleaned. Cleaning is very beneficial for both my mind and body. (DP4)”

Participants also described cognitive and emotional self-care strategies that helped them regulate emotions, process difficult experiences, and achieve psychological relief. The following excerpts illustrate these practices:

“I also engage in music. But I play my guitar for children. (DP9)”

“If there are too many counseling sessions, I avoid watching television in the evening. (DP8)”

“I do meditation. Without avoiding emotions, I accept them, knowing that others also experience these feelings. (DP1)”

“I close my eyes, lean my back against the wall, and spend 30 to 40 min contemplating. I try to make sense of my experiences in my mind and archive them. (DP6)”

“It’s not very frequent but writing among these activities makes me feel good. I sometimes write a few sentences about what I’ve experienced and then completely discard them. (DP11)”

“I draw pictures. Especially when there’s something unresolved within me, I spend hours drawing. (DP5)”

Spiritual self-care practices were reflected in participants’ efforts to maintain meaning and connection through their personal belief systems. One of the participants stated “I also try to follow the elements and rituals of my belief system. Like praying… (DP6).” Participants also emphasized the importance of interpersonal and professional support as part of their self-care. Illustrative quotations reflecting these relational and professional self-care practices are presented below:

“When I think about who I can receive support, I believe that no problem remains. We need to provide support to each other. (DP3)”

“In every situation we encounter, especially in our profession, I believe supervision is crucial. (DP3)”

##### Need for a self-care program

Regarding the need for self-care training, the majority of the participants expressed the value of a training program. As one participant expressed, “If you’re going to work in trauma, whether it’s self-care for therapists or self-care for those working in trauma, you must receive this education. Therefore, individuals working in the field should take such training in advance. (DP6),” another participant favored an in-service training by saying, “I definitely believe there should be psychoeducation related to this. It will expedite the process because yes, we all acquire knowledge during our undergraduate studies, but the professional journey unfolds differently. In our profession, we can’t always experience everything firsthand; sometimes we suddenly face situations we can’t cope with. (DP5).” On the other hand, a few remarks pointed out that such training was not necessary. One of the counselors recalled this by stating, “Being sensitive should be something that enhances self-improvement. I am against being taught in a formal course. Not everything needs a lesson. (DP3)”

Counselors also expressed a need for peer support mechanisms, such as supervision groups, debriefing sessions, or structured opportunities to share experiences with colleagues in a non-judgmental environment. In this regard, one of the counselors reported, “Experiencing things with a group feels very good. Joining self-help groups could be beneficial. (DP3)” Another key finding was the need for practical, adaptable self-care strategies that fit within the unpredictable nature of trauma work. Counselors emphasized the importance of flexible, easily integrated techniques rather than rigid self-care routines that may not be feasible in high-stress environments. Commenting on this, one participant stated, “It’s not only related to the field; self-care strategies need to be developed. Because in our daily lives, we encounter many problems. The processes we need to cope with, the thoughts we need to control—they all seem to increase over time. Rather than being caught unprepared when entering the field, it’s better to know this beforehand and proceed accordingly. (DP8)”

Overall, the needs analysis indicated that several participants perceived value in structured self-care training, peer-based support, and institutional support mechanisms. Several counselors expressed interest in learning practical strategies to support their resilience and long-term well-being in their profession.

### Study 2

#### End-session journal entry analysis results

The findings below summarize participants’ reflections on each domain following their experiences in the psychoeducation program. Figure 2 presents the themes that emerged across the five domains of self-care.

**FIGURE 2:**
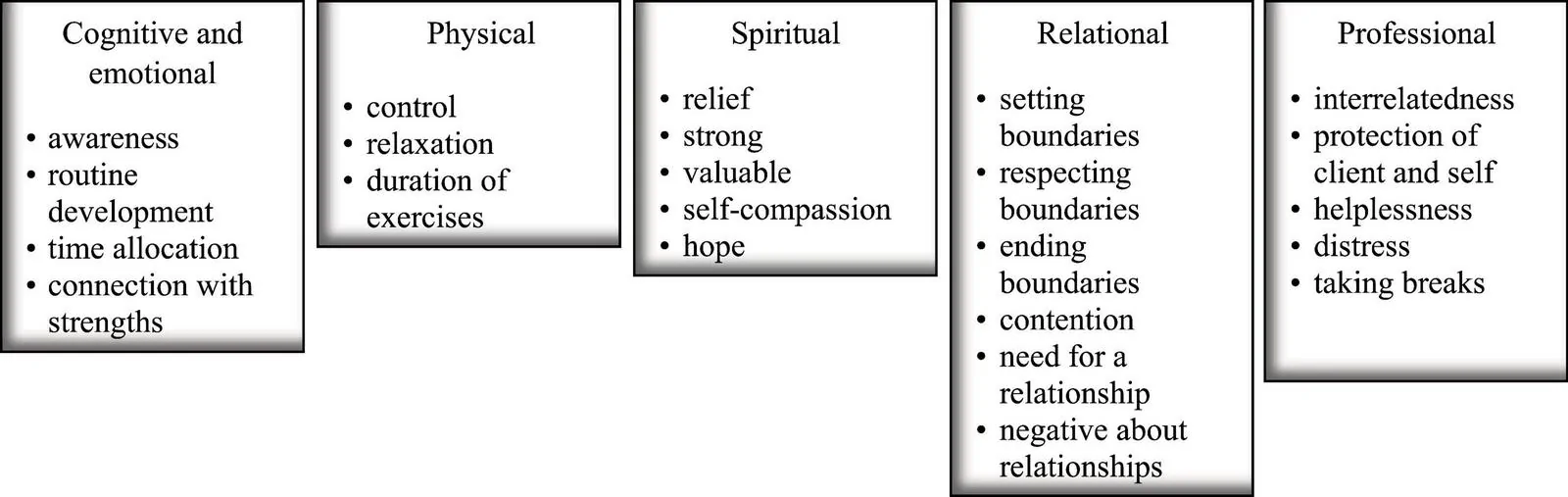
Themes pertinent to domains of self-care.

#### Findings on the domains of self-care

Across the end-session journal entries, participants reflected on different dimensions of self-care and described increased awareness of their self-care needs throughout the program. Their reflections across the cognitive-emotional, physical, spiritual, relational, and professional domains are presented below. Participants’ reflections also suggested a broader understanding of self-care following the program, extending beyond physical self-care to cognitive-emotional, spiritual, relational, and professional dimensions. This broader understanding was reflected in their awareness of different self-care needs and in their reflections on how these dimensions could be incorporated into their personal and professional lives. Participants also described increased self-awareness, particularly regarding their personal needs, emotional experiences, strengths, and self-care practices.

##### Cognitive and emotional self-care

Participants described greater awareness of compassion fatigue, the importance of establishing self-care routines, allocating personal time, and recognizing personal strengths during the cognitive and emotional self-care sessions. One participant described their awareness of strategies by stating, “I learned and experienced that compassion fatigue and burnout are normal, and there are strategies I can use for myself during these situations. (PP6),” while another comment was about the significance of developing routines in the use of strategies, “There are numerous things I want to do, but they are not routines. Facing that was slightly painful, but awareness is essential for self-care. Even though I have sufficient areas to practice self-care, I recognized the importance of developing some habits/routines in this regard… For this, I need to plan well and stick to the plan. (PP9)” Another emerging theme concerned the need to allocate time for self-care strategies, about which one interviewee commented, “I used to think I was reserving some time for myself, but this session definitely showed me that I haven’t been allocating sufficient time for myself. (PP15)” One participant specifically highlighted the clay activity as facilitating awareness of personal strengths by saying, “I would describe how the clay exercise allowed me to connect with my strengths. (PP14)”

##### Physical self-care

Participants reported that activities focusing on physical awareness and relaxation encouraged them to pay greater attention to their bodily well-being and the role of brief self-care practices in everyday life. One participant described physical awareness by stating, “Sometimes we need to turn to ourselves and physically control ourselves. That will contribute to our physical and mental health. (PP17),” while another participant commented on the relaxation exercises and their effects by saying, “Sometimes, even if it’s just 2–3 min in our lives, we need to focus on ourselves, hear our breath. This situation can allow us to turn inward personally. Breathing, our most basic need, can enable us to feel hope and our existence in life. (PP12)” Another participant cautioned about the duration of relaxation exercises by commenting, “The duration of relaxation exercises really stressed me out. (PP15)”

##### Spiritual self-care

Reflective exercises emphasizing self-compassion, personal values, and meaning-making prompted participants to describe feelings of relief, hope, and personal strength. Regarding the spiritual self-care exercises of self-compassion, worship or meditation, recalling personal values or spending time alone, the participants’ comments were “I felt relieved because I turned inward during the activities. The letter-writing activity was very meaningful. (PP7),” “I felt strong and valuable… addressing my best version was amazing. (PP5),” and “My heart warmed, I felt self-compassion, I felt hope. (PP14)”

##### Relational self-care

Activities encouraging participants to reflect on their interpersonal relationships increased awareness of personal boundaries, available sources of social support, and unmet relational needs. The interviewees emphasized setting and respecting boundaries in relationships, about which two of them commented, “I realized that my relationships are few and meaningful. I noticed areas where I need to change myself in terms of communication and respecting boundaries. (PP16)” and “I realized that boundaries are crucial for me. I am content with this situation. (PP12),” while another participant mentioned extending boundaries by saying, “I will try to break my boundaries and taboos a little more and be flexible. (PP11)” Some other participants highlighted contention in relationships by adding, “I thought I was lonelier and had fewer relationships, but I have sufficient support, warmth, understanding, love, and laughter in my life. … I realized that I should be content and start enjoying the present instead of seeking. (PP8)” One participant realized the need for an emotional relationship by stating, “I never thought about having an emotional relationship. But today, I have question marks about this. (PP10)” On the contrary, few participants had negative feelings about relationships by saying, “…having such a limited relational environment stressed me out a bit and made me question myself. (PP16)” and “However, having some relationships that I am forced to maintain and cannot completely distance myself from makes me very sad. (PP3)”

##### Professional self-care

Participants described professional self-care as extending beyond occupational competence to include personal well-being, supervision, peer learning, and healthy professional boundaries. They also reflected on the emotional challenges they experienced prior to the program and identified practical strategies they intended to implement in their professional lives. One interviewee expressed that self-care skills were interrelated by commenting, “Professional self-care is not only about occupational competence; I would also explain that it is related to other forms of self-care. (PP9),” while another one showed gratitude toward sharing different cases and solutions a trauma counselor might have by saying, “Thanks to participating and sharing experiences, I learned about cases I might encounter in my professional life and how to protect ourselves using different methods. As someone who has not yet embarked on this path, the experiences of those who have walked it before are invaluable. (PP1)”

Participants also reflected on the emotional difficulties they experienced in their professional lives and identified specific self-care practices they intended to integrate into their work. Some of the participants reflected on the negative emotions and conditions before the program by commenting, “I felt helplessness and sadness. Also, fear. I saw myself as someone who lacks the courage to pursue what I want to do. I feared not being able to recover. I also felt helpless about what I could do. (PP5)” and “I feel distress. Emotional constriction. I sense the emotional resistance that prevents me from taking action. What is holding me back? What obstacle lies in front of setting goals for myself amidst my routine and habits? (PP8)”; and, at the same time, described activities they intended to engage in to support their professional well-being by saying, “I realized that I didn’t pay enough attention to taking breaks and listening to myself while at work. (PP5),” “By taking small steps in my profession, I can make significant changes. If the activities I engage in within my field positively impact my personal life, I believe we can continue these activities. (PP10),” and “While protecting the client, it’s essential to protect ourselves too; we recognized the boundaries. (PP15)”

#### Opinions on the self-care program

The final journal entries reflected participants’ overall experiences and perceptions of the 7-week self-care psychoeducation program. Themes are illustrated in Figure 3 below.

**FIGURE 3:**
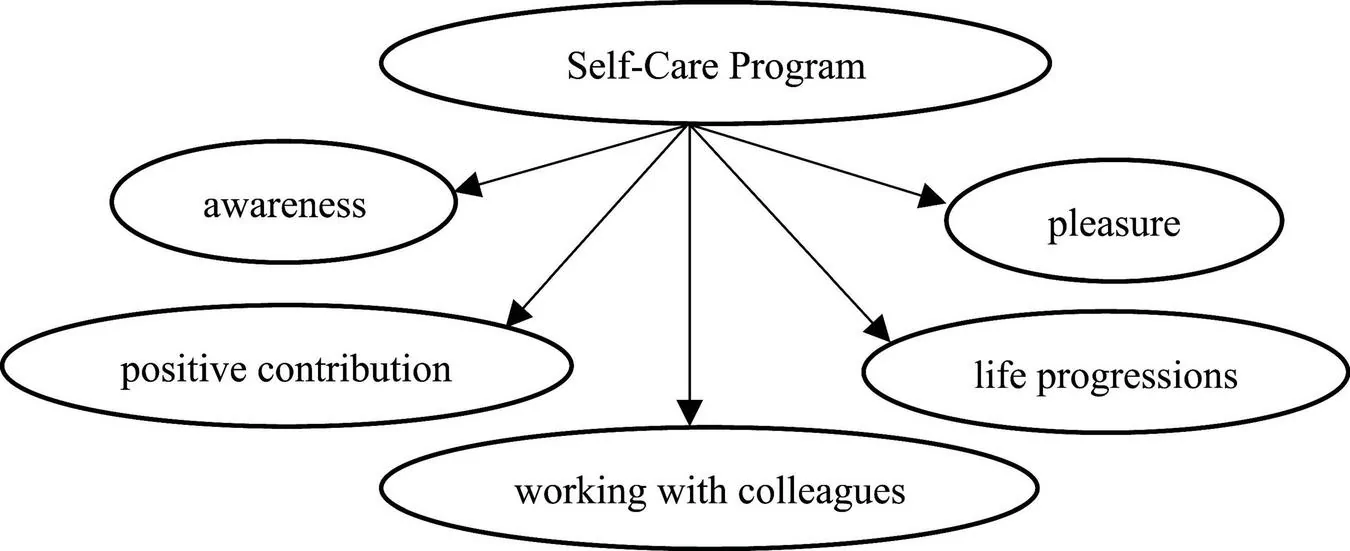
Themes regarding participant opinions.

A recurrent theme in the entries was awareness, the participants reported they gained through the program. Participants described becoming more aware of their own needs, emotional experiences, and existing self-care practices. Commenting on this, one interviewee said, “In terms of content, I believe it was very valuable. Sometimes, because we are psychological counselors and work in the field of mental health, we tend to think everything is fine. But we also have needs. In this sense, I gained awareness very well. While working in the office or at school, nobody asks how we are; they always come to us with problems. Sometimes they say, ‘You’re a psychological counselor, how can you react like this?’ We are all human, and we all have needs. Here, I regained this awareness. (PP11)” Some other comments exemplifying increase in their awareness were, “Additionally, it helped me see and become aware of my anxieties and concerns. (PP16)” and “I also noticed this. What we call self-care, we all actually do it, but being aware of it while doing it… my awareness has increased. Normally, when watching a series or movie, I used to watch just for the sake of watching, but now I’m enjoying the movie I’m currently watching. (PP1)”

Some interviewees referred to the positive contributions of the program to their daily lives, emphasizing the importance of integrating self-care practices into their routines by explicitly commenting, “Every week, we renewed ourselves and added something new. Since we did it consistently every week, toward the end, Saturdays were not the only focus; other days of the week also became the focus of my life. (PP11),” “…Procrastination doesn’t lead to results. You really need to make an effort for something. I got my violin, I’m starting my exercise routine, and I’ve already begun my project. And I’ll socialize. (PP12),” and “Lately, even after the second session, coming here has transformed into a self-care skill. I allocate time for myself, especially socialize, and experience my own awareness. (PP7)”

In their final remarks, the overwhelming majority of participants referred to pleasure of attending the program by writing comments, such as “When I started here, it was a process of turning back to myself. It was a challenging process, but now I see that I’ve come a long way. I’m very happy to be here. (PP3)” and “…but overall, I felt very good. I had a lot of fun, and no one judged each other. I opened up without any censorship, and I especially learned about self-compassion. Thank you very much; I remembered things I had forgotten. (PP6)”

The participants commented on the progress they had already made in their lives by sharing the following statements: “It also contributed to my own progress. The activities here were similar to the assignments given by my therapist, which made progress a bit easier for me there. (PP9),” “My motivation was low when faced with things I needed to do. I knew what I had to do, but I had no motivation for them. After coming here, I started finding sources of motivation within myself. (PP16),” “I needed socialization, sharing, and learning. This education fulfilled all of these. (PP15),” and “In the section where I talked about the problems I experienced, being part of a group with similar qualities made me realize that I am not alone. It felt good to know that I share positive attributes with others. (PP9)”

The benefit of sharing the same setting with other colleagues can be observed in their recollections reporting, “Here, everyone shares their own self-care activities, different things. Hearing things I’ve never heard before, things that are good for everyone, was very beneficial for me. (PP8),” “Because I encountered very judgmental people in the field, the acceptance of my colleagues here, without judgment, criticism, and their ability to share their thoughts, provided me with incredible comfort…(PP3),” and “It was also wonderful for me that different colleagues shared their thoughts and experiences. (PP7)”

## Discussion

The primary aim of this study was to develop and pilot a multidimensional self-care psychoeducation program for trauma counselors and to explore participants’ experiences during its implementation.

### The development of the program

The needs analysis conducted in the preliminary phase of this study provided a rich, qualitative understanding of the experiences and expectations of trauma counselors. Participants frequently reported emotional exhaustion, intrusive thoughts, numbness, and difficulties in emotional regulation as consequences of prolonged exposure to clients’ trauma narratives. These findings are consistent with previous literature on secondary traumatic stress (STS), vicarious trauma, and burnout (Figley, 1995; Maslach and Leiter, 2016; Pearlman and Saakvitne, 1995b). Witnessing intense emotional pain, trauma counselors may internalize these experiences, which have been associated with a breakdown in emotional resilience and professional functioning (Adams et al., 2006; Silveira and Boyer, 2015).

The participants’ comments on significant institutional and cultural barriers to self-care, including unrealistic workloads, insufficient supervision, and an organizational ethos that prioritizes client care at the expense of practitioner well-being reflect the existing critiques of mental health systems that fail to adequately support clinicians’ needs (Corey, 2013; Newell and MacNeil, 2010). Although many participants reported employing informal strategies such as exercise, mindfulness, and social engagement, these efforts were described as inconsistent and often insufficient in the face of ongoing work-related stress. These findings are consistent with previous studies suggesting that without structured guidance, self-care remains underutilized and poorly integrated into professional practice (Norcross and Guy, 2018; Posluns and Gall, 2020).

The desire for peer support, debriefing sessions, and a more compassionate workplace culture emerged as another major theme. Participants expressed the importance of feeling validated and understood by their colleagues, a need that has been previously linked to reduced burnout and improved professional satisfaction (Barlow and Phelan, 2007; Merriman, 2015). Moreover, the need for flexible, personalized, and context-sensitive self-care strategies emerged as a salient theme. Similarly, Posluns and Gall (2020) emphasized that self-care must be both adaptable and holistic to be sustainable. Counselors expressed the desire for peer-based support structures such as debriefing groups and professional supervision, which reflected the findings of Barlow and Phelan (2007) and Merriman (2015), who stressed the importance of collegial connection and supervision in preventing compassion fatigue.

Participants’ narratives suggested that many perceived self-care extending beyond individual coping strategies and highlighted the importance of organizational support. This interpretation is consistent with previous literature emphasizing the role of organizational responsibility in promoting practitioner well-being (Dreison et al., 2018; Pearlman, 1999).

Nevertheless, the findings from the needs analysis informed the development of the psychoeducation program and shaped its experiential, multi-dimensional structure, designed specifically to address the realities of trauma professionals in Turkiye. These findings suggest a perceived need among participants for more structured approaches to counselor well-being within professional training and organizational settings. In light of the needs expressed in the preliminary analysis and supported by extant literature on self-care, the psychoeducation program was designed specifically to include practical content, guidance on integrating strategies into daily life, peer interaction through shared experiences, post-session materials, and daily journaling.

#### Experiences gained through piloting

The psychoeducation program designed for this study aimed to respond directly to the needs identified in the initial analysis. While pre-intervention responses often equated self-care with physical maintenance, post-intervention reflections expanded to include emotional, cognitive, spiritual, relational, and professional dimensions. These findings appear consistent with multidimensional approaches to self-care described in previous literature which emphasize the need for a holistic approach (Culver, 2011; Godfrey et al., 2011; Posluns and Gall, 2020). One notable theme emerging from the participants’ reflections was increased self-awareness. Participants described becoming more attuned to their emotional states, professional boundaries, and personal needs. This finding appears consistent with the conceptualization of self-care as a self-regulatory and preventive practice (Coster and Schwebel, 1997; Park, 2010).

Participants reported their intentions to engage more actively in self-care routines, rather than passive or reactive practices, which is considered a core feature of effective self-care (Lee and Miller, 2013). Group-based learning emerged as a valued component of the program in participants’ reflections. Participants consistently highlighted the value of shared experiences, emotional safety, and mutual learning. These findings are consistent with previous research on the role of community and peer support in fostering professional resilience (Livanou et al., 2024; Merriman, 2015). Participants described the group setting as providing a sense of normalization, openness, and nonjudgmental sharing (Grandey, 2000). Participants also associated the interactive structure of the program with opportunities for peer-based reflection and shared learning.

Participants described the experiential activities as facilitating personal reflection and helping them relate self-care strategies to their own personal experiences. This observation is consistent with literature suggesting that experiential and emotionally engaging methods may facilitate reflection and engagement in counselor training (Craig and Sprang, 2010; Warren et al., 2010). Finally, several participants identified a shift in their perception of self-care, not as a luxury, but as a vital and ethical element of their professional identity. This aligns closely with American Counseling Association [ACA] (2014) and Canadian Counseling and Psychotherapy Association [CCPA] (2020) ethical standards, which frame self-care as a responsibility rather than an option. Participants’ reflections suggested that many came to view self-care as an internal aspect of their personal identity. This interpretation is consistent with ethical frameworks that position self-care as an important professional responsibility. However, these findings represent participants’ subjective experiences following the pilot implementation of the program.

### Participants’ reflections on multidimensional self-care

Trained trauma counselors described increased awareness of the multidimensional structure of self-care following their participation in the pilot program. They reported becoming more aware of thoughts, emotions, and behaviors that they perceived as limiting their self-care practices. They also described reflecting on ways to incorporate self-care strategies into their daily lives.

Participants reflected on employing cognitive and emotional self-care strategies, including pause techniques, journal writing or storytelling, enhancing leisure activities, reading books, playing musical instruments, participating in artistic endeavors, working with clay, solving puzzles, and even taking singing lessons to cope with work-related stress. Additionally, they expressed their intention to connect more with colleagues to share experiences and incorporate this into their self-care strategies for cognitive relaxation. Participants further described becoming more attentive to their personal strengths, immediate needs, and emotional experiences, a finding that is consistent with previous discussions of self-awareness in self-care (Jiménez-Giménez et al., 2021). They emphasized the significance of allocating time and personal space for oneself and integrating all these strategies into their lives in a more sustainable and organized manner.

Previous research suggests that physical self-care strategies are used within other dimensions of self-care, and in fact, they encompass cognitive, emotional, and other dimensions (Schure et al., 2008). Research on the effects of physical activity on depression and burnout indicates that individuals with moderate to high fitness levels report fewer symptoms of burnout and depression compared to those with low fitness levels (Gerber et al., 2013). Supporting this, Thériault et al. (2015) highlight the importance of prioritizing physical health and well-being and engaging in relaxation exercises as coping strategies for MHPs. Additionally, Greenwood and Fleshner (2008) emphasize that physical exercise prevents stress-related illnesses. Having previously neglected physical activity, participants expressed their intention to take steps toward physical self-care, including exercising, walking, starting or continuing breathing exercises, meditation, or yoga, regulating sleep hours, maintaining a regular and healthy diet, initiating or continuing individual hobbies, paying attention to physical appearance, scheduling regular health check-ups, taking breaks and resting, and spending time with pets.

Participants also emphasized the importance of inner dialogue and the ability to listen to their inner voice, aiming to establish stronger communication with themselves, the result of which is consistent with related studies emphasizing the significance of inner dialogue in communication (Moore, 2013). Self-compassion, which is considered to specifically reduce stress among psychological counselors (Shapiro et al., 2007), was another spiritual self-care strategy well received by the participants. Though not frequently engaged in before the program, the participants came to recognize the importance of spiritual self-care for well-being and they reported intending to implement some of the strategies, such as re-examining the meaning of life, approaching themselves with self-compassion, recognizing their strengths, establishing a connection with a perceived higher power through worship or meditation, seeking new perspectives from movies, books, or series, gaining self-awareness, remembering personal values, spending time alone, and taking breaks. Additionally, participants emphasized that experiencing spiritual self-care strategies alongside all this newfound awareness evoked positive emotions. These reflections are consistent with previous studies in which spiritual self-care strategies have been associated with optimism, wisdom, life satisfaction, and self-compassion (Nelson et al., 2018).

Within the context of relational self-care, the participants realized that they didn’t dedicate enough time to their loved ones and those who provided them with social support. They acknowledged that they had neglected these relationships and needed to review their connections, needs, and boundaries. Additionally, participants noted that they did not sufficiently exhibit functional behaviors such as directly expressing their emotions, avoiding excessive responsibility, saying no to requests they were unwilling to fulfill, maintaining realistic and functional expectations, and managing their boundaries. Similarly, Thériault et al. (2015) highlight the significance of social support, especially for psychological counselors in coping with problems and Myers et al. (2012) report stress-reducing effects of social support.

For professional self-care, there was a focus on sharing experiences related to understanding and interpreting individual challenges, learning new strategies to balance professional and personal lives, and internalizing the concept of balance. Participants discussed intentions to continue their professional development, improve time management, and to make greater use of integrated supervision and consultation as part of their ongoing self-care. These reflections are consistent with studies highlighting the value of effective education, along with supervision, continuous professional development, and organizational support in reducing secondary trauma symptoms in MHPs (Harrison and Westwood, 2009; Thériault et al., 2015).

### Implications

As an exploratory qualitative pilot study, the present study offers preliminary implications for intervention development, psychological counselor education programs, and future research. These implications should be interpreted in light of the exploratory nature of the study and participants’ subjective experiences. Beginning with the implications for clinical practice, participants described increased awareness of self-care practices following the multidimensional psychoeducation program and reflected on their personal needs, coping strategies, and self-care behaviors. The participants’ increased engagement in self-reflective behaviors, use of personalized coping strategies, and acknowledgment of self-care as an ethical imperative suggest that this type of intervention may represent a promising avenue for supporting trauma counselors. However, further research is needed before conclusions can be drawn regarding their effectiveness. Although participants perceived peer interaction, reflective activities, and experiential learning as valuable components of the program, additional research using controlled and longitudinal designs is needed before recommending broader implementation in organizational settings.

Secondly, considering the implications for counselor education and program development which are grounded in the need to revise psychological counseling curricula in Turkiye, the authors suggest incorporating structured self-care education at both undergraduate and graduate levels. Despite the recognition of burnout and secondary trauma in the literature, self-care training remains limited in formal education. Council for Accreditation of Counseling and Related Educational Programs [CACREP] (2016), American Counseling Association [ACA] (2014), and Canadian Counseling and Psychotherapy Association [CCPA] (2020) ethical codes support such integration, reinforcing that self-care is not just a best practice, but a professional duty. While Güler (2018) proposed a self-care training module for undergraduate students, and the current study extended this work by targeting in-service professionals, the present findings further highlight the need for additional research examining self-care education that addresses the well-being of counselors working in the field of trauma.

Finally, as an implication for future research, longitudinal studies should investigate whether the reported changes in self-awareness and self-care practices are sustained over time. Moreover, it is also necessary to examine the role of individual differences (e.g., trauma history, gender, and years of experience) and institutional factors (e.g., caseload, support systems) in moderating the effectiveness of psychoeducation programs.

This study has several limitations that should be considered when interpreting the findings. As an exploratory qualitative pilot study, the findings reflect participants’ subjective experiences following the implementation of the psychoeducation program and should not be interpreted as evidence of intervention effectiveness. The needs assessment and psychoeducation protocol were developed through a literature-informed, expert-reviewed, and pilot-tested process; however, given the exploratory nature of the study, these developmental procedures do not constitute formal validation of the needs-assessment instrument or the intervention protocol. In addition, the needs-assessment interview protocol was piloted with only two trauma counselors; although this pilot supported refinement of the questions, the small number of pilot participants limited the breadth of feedback available for assessing the protocol. The relatively small sample size and the development and piloting of the program within the specific sociocultural and institutional context of Turkiye may limit the transferability of the results to different cultural or organizational settings. Second, the qualitative design, reliance on self-report journal entries, and the absence of baseline measures or a comparison group may have introduced social desirability bias and restricted the ability to draw causal inferences. Formal intervention fidelity assessment was not conducted in this exploratory qualitative pilot; therefore, the extent to which session delivery was consistent across implementation cannot be independently evaluated. Furthermore, participants’ reported experiences may also have been influenced by factors such as peer interaction, group cohesion, facilitator influence, increased reflective practice, or expectancy effects rather than the psychoeducation program alone. Finally, as participants were interviewed immediately following the seven-week program, the absence of longitudinal follow-up makes it difficult to determine whether these reported experiences were sustained over time. Future research employing longitudinal, controlled, and mixed-method designs is needed to further examine the effectiveness and longer-term impact of multidimensional self-care psychoeducation programs.

## Conclusion

This exploratory qualitative pilot provides preliminary insights into trauma counselors’ experiences of participating in a multidimensional self-care psychoeducation program developed in response to their identified needs. Grounded in qualitative needs analysis and existing self-care literature, the psychoeducation program was designed to address the emotional, cognitive, physical, spiritual, relational, and professional dimensions of self-care for trauma counselors practicing within the unique sociocultural context of Turkiye. Participants described increased awareness of multidimensional self-care and reflected on the importance of integrating self-care into their personal and professional lives. Many participants also described viewing self-care as an important aspect of their professional identity following the pilot program. Group-based experiential learning, reflective practices, and peer support were identified as valuable components of the program and discussed in relation to their reflections on self-care strategies. Participants’ reflections further suggested a broader understanding of self-care, extending beyond individual practices to include relational, professional, and organizational dimensions. Overall, these preliminary findings contribute to the growing literature on self-care among trauma counselors and provide a foundation for the further development and evaluation of multidimensional self-care psychoeducation programs.

## Data Availability

The qualitative data generated and/or analyzed during the current study are not publicly available because they contain potentially identifiable information from a small group of mental health professionals discussing trauma-related experiences. The data may be made available by the corresponding author upon reasonable request, subject to ethical approval, participant consent, and appropriate measures to protect against deductive identification.
